# SaLT&PepPr is an interface-predicting language model for designing peptide-guided protein degraders

**DOI:** 10.1038/s42003-023-05464-z

**Published:** 2023-10-24

**Authors:** Garyk Brixi, Tianzheng Ye, Lauren Hong, Tian Wang, Connor Monticello, Natalia Lopez-Barbosa, Sophia Vincoff, Vivian Yudistyra, Lin Zhao, Elena Haarer, Tianlai Chen, Sarah Pertsemlidis, Kalyan Palepu, Suhaas Bhat, Jayani Christopher, Xinning Li, Tong Liu, Sue Zhang, Lillian Petersen, Matthew P. DeLisa, Pranam Chatterjee

**Affiliations:** 1 Department of Biomedical Engineering, Duke University, Durham, NC USA; 2 Robert F. Smith School of Chemical and Biomolecular Engineering, Cornell University, Ithaca, NY USA; 3 Meinig School of Biomedical Engineering, Cornell University, Ithaca, NY USA; 4 Cornell Institute of Biotechnology, Cornell University, Ithaca, NY USA; 5 Department of Computer Science, Duke University, Durham, NC USA; 6 Department of Biostatistics and Bioinformatics, Duke University, Durham, NC USA

**Keywords:** Recombinant protein therapy, Protein design

## Abstract

Protein-protein interactions (PPIs) are critical for biological processes and predicting the sites of these interactions is useful for both computational and experimental applications. We present a **S**tructure-**a**gnostic **L**anguage **T**ransformer and **Pep**tide **Pr**ioritization (SaLT&PepPr) pipeline to predict interaction interfaces from a protein sequence alone for the subsequent generation of peptidic binding motifs. Our model fine-tunes the ESM-2 protein language model (pLM) with a per-position prediction task to identify PPI sites using data from the PDB, and prioritizes motifs which are most likely to be involved within inter-chain binding. By only using amino acid sequence as input, our model is competitive with structural homology-based methods, but exhibits reduced performance compared with deep learning models that input both structural and sequence features. Inspired by our previous results using co-crystals to engineer target-binding “guide” peptides, we curate PPI databases to identify partners for subsequent peptide derivation. Fusing guide peptides to an E3 ubiquitin ligase domain, we demonstrate degradation of endogenous β-catenin, 4E-BP2, and TRIM8, and highlight the nanomolar binding affinity, low off-targeting propensity, and function-altering capability of our best-performing degraders in cancer cells. In total, our study suggests that prioritizing binders from natural interactions via pLMs can enable programmable protein targeting and modulation.

## Abstract (web summary)

SaLT&PepPr is a protein language model that isolates peptidic motifs from the binding interfaces of target-interacting partner sequences. These peptides are fused to an E3 ligase domain to generate peptide-guided protein degraders for diverse targets without structure.

## Introduction

**Fig. 1:**
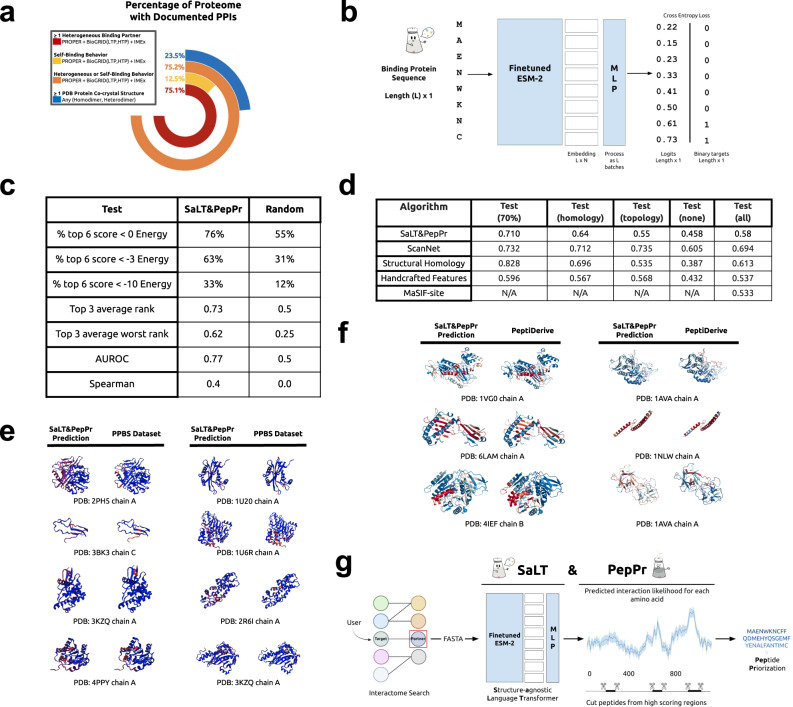
An interface-predicting language model for peptide prioritization. **a** Percent of proteome with known interacting partners, characterized by known structural/sequence information. Partner data was calculated from the IMEX, BioGRID, and PROPER databases. LTP and HTP refer to the either “low throughput” or “high throughput” physical evidence for an interaction in BioGRID. Co-crystal statistics were derived from the RCSB PDB. **b** The SaLT model is based on ESM-2 with a multilayer perceptron (MLP) classification head, trained to predict the probability of an amino acid position being a protein binding site. **c** Model performance was evaluated on a held out, nonhomologous test set, established from PeptiDerive. Energy units refer to averaged REU per amino acid position. **d** Benchmarking of SaLT&PepPr trained on PPBS and then tested on different test splits. PPBS dataset, test splits, as well as model test scores are obtained from Tubiana et al.^12^ The PPBS dataset splits represent: 70% (at least 70% sequence homology with one training example), homology (at most 70% homology with a train set example, although at least one train set belongs to the same protein superfamily), topology (at least one train set has a similar protein topology with none in the similar protein family) and None (none of the above groups). The structural homology baseline uses template protein chains with known binding sites, a local pairwise structural comparison, and an alignment weighting scheme. The handcrafted features baseline includes 58 features based on the structural, atomic, and sequence information, with an XGBoost algorithm. Note that MaSIF-site was not retrained for the per-residue task. **e** Comparison between the predicted SaLT&PepPr scores and experimentally-annotated PPBS binding sites on different protein structures in the PPBS dataset. Red indicates high binding probability amino acids, with blue as low binding probability, normalized for each protein chain. **f** Representative examples of model inference versus calculated PeptiDerive energy landscapes from specific PDB co-crystal entries. Red indicates high binding probability, with white as lower and blue as low, and gray indicates amino acids which are discarded because of being invalid for PeptiDerive. Note that PeptiDerive scores visualized only reflect binding sites captured in the specific PDB entry. **g** To predict binding peptides, partner proteins are identified using an interactome search, and are then passed as input to the model. The graph represents the probability of each position being a PPI site, the output of our model. Continuous sequences of amino acids are cut from the sequence along the curve, which can be done algorithmically or with user judgment.

Fusing compact protein binders to various E3 ubiquitin ligase domains enables selective binding, ubiquitination, and intracellular degradation of diverse proteins of interest^1–4^. Generating a modular system to design these genetically encoded constructs, termed ubiquibodies (uAbs), will represent a flexible approach for targeted protein degradation (TPD). Inspired by the programmability of RNA-guided CRISPR genome editing^5^, in recent work, we have previously used linear motifs identified from the binding interfaces of bound co-crystal structures of protein-protein interactions (PPIs) to serve as “guide” peptides for subsequent generation of target-degrading uAbs^2^. However, our structure-based method relies on experimentally-validated co-crystals of target proteins, which exist for only <25% of the human proteome (Fig. 1a). Gold-standard PPI databases contain binder sequences to over 75% of the human proteome, and thus represent a rich source of information for guide peptide generation (Fig. 1a)^6–8^. As such, we hypothesize that leveraging PPI information to identify protein interaction sites from the sequence of a partner protein may enable more broad-scale prioritization of guide peptides for uAb-mediated TPD.

In this work, we apply protein language models (pLMs) to identify binding motifs from input protein sequences, without the requirement of three-dimensional protein structures. By accurately predicting these protein binding sites on verified interacting partners, we prioritize guide peptides for downstream uAb generation. To do this, we create a **S**tructure-**a**gnostic **L**anguage **T**ransformer **& Pep**tide **Pr**ioritization (SaLT&PepPr) model based on the state-of-the art ESM-2 pLM^9^, that first predicts the interaction sites along an input interacting partner sequence, and via integration with PPI databases, enables isolation of continuous guide peptide candidates for an input target protein. As a first proof-of-concept, we leveraged known interaction information to generate high-affinity, specific peptide-guided degraders of β-catenin, a core transcriptional regulator whose dysregulation frequently leads to cancer cell proliferation^10^. We then showed that SaLT&PepPr can effectively prioritize guide peptides to 4E-BP2 and TRIM8 in a data-driven manner, which were integrated into the uAb architecture and found to induce target degradation. TRIM8-targeting uAbs, specifically, induced apoptosis in Ewing sarcoma cells, in line with previous genetic studies^11^. In total, our work showcases how integrating natural protein interactions, binding site prediction via pLMs, and genetically-encoded protein constructs enables the rapid generation of uAbs for modular TPD applications.

## Results

### Fine-tuned language model for interface prediction

We trained a model to predict interaction sites on a given partner sequence for a target protein. We based our model architecture on the 650-million parameter ESM-2 pLM, from Meta AI, which enables featurization of protein sequences without the need for multiple sequence alignment (MSA) generation^9^. We fine-tuned the final three layers of ESM-2 together with a multilayer perceptron (MLP) classification head, where each protein sequence was passed to the model with a per amino acid binary class as the target, employing a binary cross entropy loss (Fig. 1b). We trained and assessed our model separately on two datasets of PPI sites: the protein-protein binding site (PPBS) dataset from ScanNet and our own PPI site data, derived using the PeptiDerive method on co-crystal structures in the PDB (Supplementary Fig. 1)^12,13^. For both datasets, a nonhomologous test and validation set were used to assess the generalization of the model in predicting interaction hotspots.

Overall, SaLT&PepPer exhibited robust performance on non-homologous validation and test sets, demonstrating generalizability of our model. When trained and tested on the PDB-derived dataset, SaLT&PepPr achieved a test set area under the ROC curve (AUROC) of 0.77 (Fig. 1c). Alternatively, when keeping ESM-2 weights frozen, the test AUROC was 0.7, demonstrating the benefit of fine-tuning the final layers of the original model. This approach, which utilizes the sequence of the binding partner, has a Spearman correlation to PeptiDerive energy scores of 0.4 on the test set with sequence homology <25% (Fig. 1c). We also trained and tested on the ScanNet PPBS dataset to compare our model to baseline and state-of-the-art models which require tertiary structure and/or multiple sequence alignments (MSAs) to identify protein interacting residues^12,14^. Despite not using structure as input, our model achieved competitive performance compared to structure-based benchmarks, and decreased performance compared to ScanNet (Fig. 1d). Specifically, on the “Test none” split which reflects most distant proteins, SaLT&PepPr exhibited superior performance to baseline methods based on structural homology and handcrafted feature selection, suggesting strong generalization to non-homologous proteins from different families (Fig. 1d). Finally, we visualized SaLT&PepPr predictions on partner proteins with available crystal structures from the PDB, highlighting the model’s capability to identify concise interacting interfaces, both in isolated structures of single binding proteins (Fig. 1e) and within co-crystals (Fig. 1f).

### Peptide prioritization using predicted interfaces on binding partners

Previously, we demonstrated the utility of using interacting partners to derive functional peptides for uAb generation by executing the Rosetta-based PeptiDerive protocol on existing co-crystals containing the target protein, identifying the linear polypeptide segments suggested to contribute most to binding energy^2^. However, this method relies on experimentally-derived co-crystals, which only capture a small percentage of the ~650,000 known human protein-protein interactions (PPIs)^15^. To ameliorate this shortcoming, we integrated our model with available PPI datasets to identify linear peptide guides for a given target protein by predicting the binding sites on partners that interact with the target of interest (Fig. 1a)^6–8^. As a result, peptides can be sampled across the partner sequence to both maximize breadth of selection or incorporate prior knowledge of known target-selective binding domains. Specifically, as our model predicts the probability of each amino acid in the partner protein sequence being an interaction site, continuous peptides were “cut” from the full partner sequence via a local-maximum sampling approach to isolate peptides of a user-specified length with highest predicted probability of binding that also represent local maxima in the predicted binding likelihood (Fig. 1g). In total, the inference time for a single target protein in SaLT&PepPr took about one minute on a standard machine with 2 CPU cores, 8 GB of RAM, and no GPU, far more efficient than methods requiring structure prediction (Supplementary Fig. 2)^16,17^.

### Characterization of interface-derived uAbs for targeted β-catenin degradation

**Fig. 2:**
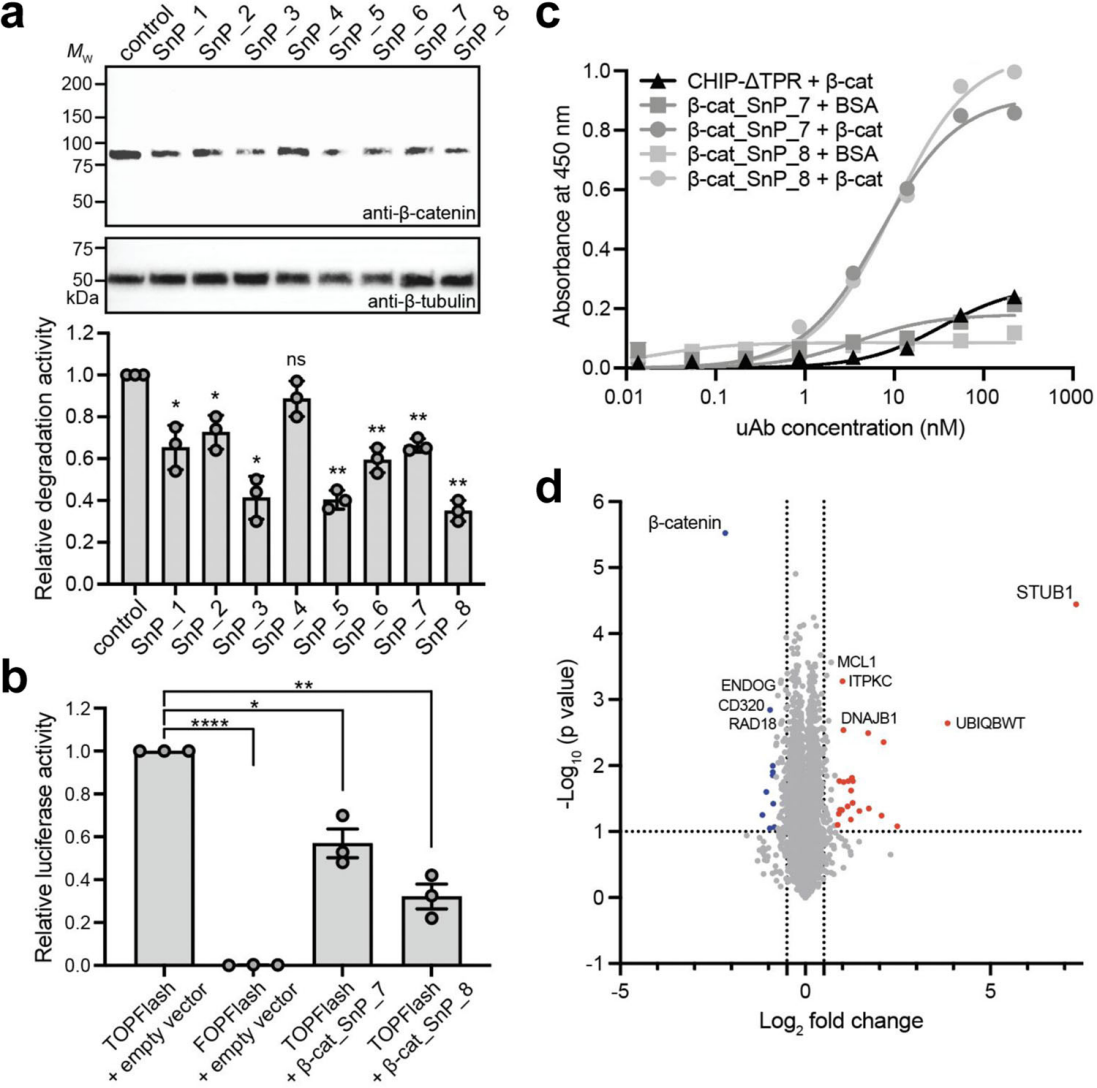
Characterization of peptide-guided uAbs for targeted β-catenin degradation. **a** Degradation of endogenous β-catenin in cytosolic fraction of DLD1 cells analyzed via immunoblotting with anti-β-catenin and anti-β-tubulin antibodies. Blots are representative of independent transfection replicates (*n* = 3). Relative degradation activity was determined by densitometry analysis of anti-β-catenin immunoblot. **b** TOPFlash luciferase reporter assay of β-catenin/TCF transcriptional activity. FOPFlash reporter served as negative control. **c** β-catenin binding activity determined by ELISA with immobilized β-catenin (β-cat). Binding to bovine serum albumin (BSA) served as negative control. **d** Nano LC-MS/MS analysis of total proteins collected from HEK293T cells co-transfected with plasmids encoding SnP_8 uAb and β-catenin-sfGFP. Data were log_2_-normalized and fold-change and *p*-value (unpaired, two-tailed *t*-test) were performed to generate volcano plot of differentially abundant proteins. STUB1 denotes overexpressed CHIPΔTPR domain of SnP_8 uAb. Data in **a**–**c** are the average of independent transfection replicates (*n* = 3) ± SD. For individual samples, statistical significance was determined by two-tailed Student’s *t* test.

Recently, based on the seminal work of Portnoff et al.^1^, our group reprogrammed the specificity of a modular human E3 ubiquitin ligase called CHIP (carboxyl-terminus of Hsc70-interacting protein) by replacing its natural substrate-binding domain, TPR, with designer “guide” peptides to generate minimal and programmable uAb architectures^2,18,19^. To demonstrate that guide peptides derived from known, selected interacting partners can function as robust guide peptides, we first focused on designing uAbs to β-catenin, as aberrant Wnt/β-catenin signaling is widely implicated in numerous cancers, including colorectal, hepatocellular, lung, and pancreatic^10,20^. Specifically, mutated β-catenin accumulates in the cytosol of affected cells, while wild-type β-catenin binds to the transmembrane protein, E-cadherin^20^. Thus, to enable degradation of endogenous, cytosolic β-catenin, we leveraged its known sequence interaction with E-cadherin to select guide peptides from the E-cadherin/β-catenin binding interface for subsequent uAb generation, and scored them with SaLT&PepPr (Supplementary Table 1)^21^. We then transfected DLD1 colon cancer cells, which express wild-type β-catenin at abnormally high levels, with our uAb constructs (SnP_1 to SnP_8). Immunoblots of the cytosolic fractions revealed that all but one uAb promoted statistically significant β-catenin degradation relative to non-transfected DLD1 control cells, with several (SnP_3, SnP_5, SnP_8) degrading >60% of the cytosolic β-catenin pool (Fig. 2a). Using TOPFlash^22^, a luciferase reporter that serves as a reliable readout of β-catenin–dependent transcriptional activity, we observed that the strong SnP_8 degrader dramatically decreased the transcriptional response to β-catenin relative to empty vector control cells (Fig. 2b). For comparison, the SnP_7 degrader induced a more modest inhibitory effect on β-catenin signaling, in line with its intermediate degradation activity.

We confirmed that peptide-guided uAbs promoted target degradation through specific, peptide-mediated binding of β-catenin as demonstrated by quantitative ELISA (Fig. 2c). Specifically, purified versions of SnP_7 and SnP_8 uAbs exhibited strong affinity to immobilized β-catenin with virtually no binding to the immobilized bovine serum albumin (BSA) control. The strong β-catenin binding exhibited by SnP_7 and SnP_8 was attributable to the SaLT&PepPr peptides as evidenced by the lack of binding for the CHIPΔTPR ubiquitination domain alone. We note that the relatively high binding activity of these uAbs for β-catenin was in line with the binding affinity measured for other uAbs^1,23^. Given the similar binding activity yet different levels of β-catenin silencing, other factors such as proximity/orientation upon binding must also contribute to the efficacy of peptide-guided uAbs.

Finally, to test the off-targeting propensity of our peptide-guided uAbs, one dimensional liquid chromatography-tandem mass spectrometry (1D-LC-MS/MS) analysis was performed on total proteins harvested from cells overexpressing β-catenin, with or without treatment with the uAb candidates, with ~6700 proteins were quantified. Our analysis demonstrated the expected increase in uAb-associated proteins, including tryptic peptides assigned to the human CHIP protein (STUB1), and a corresponding decrease in β-catenin abundance between the control and treated samples for both tested uAbs (Fig. 2d and Supplementary Fig. 3). In contrast, there were no significant changes in the abundance of other proteins as a function of uAb expression, confirming that there were no statistically significant off-target effects associated with uAb expression or degradation.

### Experimental validation of SaLT&PepPr interface prediction for endogenous target degradation

**Fig. 3:**
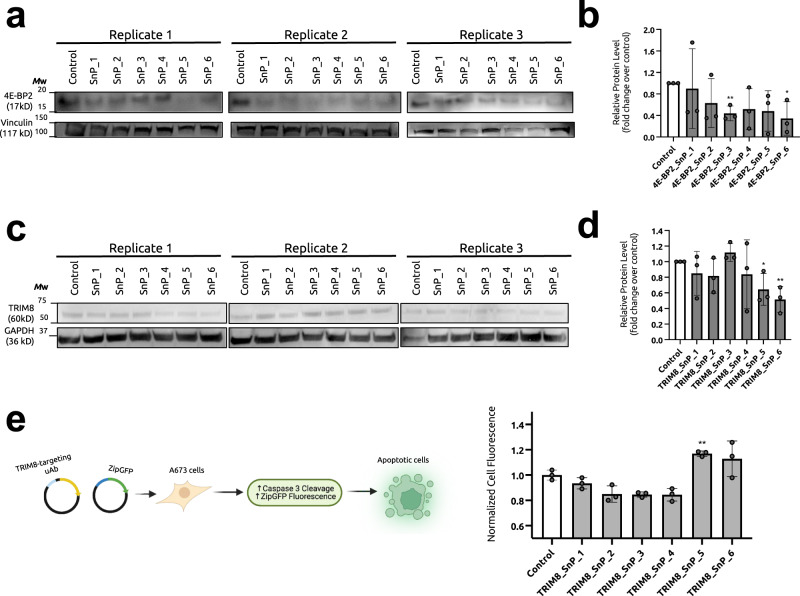
Characterization of SaLT&PepPr-derived uAbs for endogenous target degradation. **a** Degradation of endogenous 4E-BP2 in protein extracts of A673 cells analyzed via immunoblotting with anti-4E-BP2 and anti-Vinculin antibodies. Blots are representative of independent transfection replicates (*n* = 3). **b** Relative degradation activity was determined by densitometry analysis of 4E-BP2 signal normalized to sample-specific Vinculin signal. For individual samples, statistical significance was determined by a two-tailed Student’s *t* test to the non-targeting control. Calculated *p* values are represented as follows: *p < 0.05; **p  <  0.01. **c** Degradation of endogenous TRIM8 in protein extracts of A673 cells analyzed via immunoblotting with anti-TRIM8 and anti-GAPDH antibodies. Blots are representative of independent transfection replicates (*n* = 3). **d** Relative degradation activity was determined by densitometry analysis of TRIM8 signal normalized to sample-specific GAPDH signal. For individual samples, statistical significance was determined by a two-tailed Student’s *t* test to the non-targe*t*ing control. Calculated *p* values are represented as follows: *p  <  0.05; **p  <  0.01. **e** For the apoptosis assay (shown in the schematic), A673 cells were co-transfected with equal ratios of the uAb plasmid and ZipGFP caspase reporter plasmids. Cells expressing mCherry (transfection reporter) were gated, and normalized EGFP cell fluorescence, indicating functional reporter activity, was calculated to samples transfected with a non-targeting uAb. For individual samples, statistical significance was determined by a two-tailed Student’s *t* test to the non-targeting control. Calculated *p* values are represented as follows: **, *p* < 0.01.

Having established the ability to use interacting partners as effective scaffolds for guide peptide generation, we sought to test SaLT&PepPr’s ability to prioritize effective guide peptides in a data-driven manner. To do this, we first chose eukaryotic initiation factor 4E binding protein 2 (4E-BP2), a relatively small and disordered protein involved in eukaryotic translation initiation that has also been implicated in cancer^24,25^. 4E-BP2 has a single known specific interactor: eukaryotic initiation factor 4E (eIF4E)^26^. Using the eIF4E as input into SaLT&PepPr, we derived the top six high-scoring peptides from its sequence (Supplementary Table 1). These peptides were cloned into our uAb plasmids, and transfected into A673 Ewing sarcoma cells, where 4E-BP2 is highly expressed. Following Western blotting post treatment, we successfully identified two degraders, 4E-BP2_SnP_3 and 4E-BP2_SnP_6, demonstrating over 50% degradation of endogenous 4E-BP2 as compared to that of a non-targeting control plasmid (Fig. 3a-b), highlighting the utility of our algorithm.

We next turned our focus to TRIM8, an E3 ubiquitin ligase itself that regulates the levels of the core fusion oncoprotein driving Ewing sarcoma, EWS-FLI1^11^. Loss of TRIM8 induces EWS-FLI1-mediated overdose in Ewing sarcoma cells, leading to upregulation of apoptosis^11^. Using TRIM8 as an input into our curated PPI database to identify multiple interacting partners (Fig. 1a), we used SaLT&PepPr to derive the top six highest-scoring peptides from various partners and integrated them into our uAb architecture (Supplementary Table 1). Next, we transfected these uAbs into A673 Ewing sarcoma cells, and successfully identified two candidates, TRIM8_SnP_5 and TRIM8_SnP_6, that degraded endogenous TRIM8 with statistical significance (Fig. 3c, d). We then co-transfected these six uAbs alongside a GFP-based fluorogenic caspase reporter of apoptosis in A673 cells, termed ZipGFP^27^, and observed that our most effective degraders induced upregulation of apoptosis, as expected from previous studies (Fig. 3e)^11^.

## Discussion

Together, our results suggest a method to degrade proteins in a CRISPR-analogous manner by identifying binding sites from natural protein interactions without the use of structural information. Fusing these partner protein-derived guide peptides to E3 ubiquitin ligase conjugation domains yields a simple, genetically-encoded uAb architecture for downstream TPD. While we demonstrate success on β-catenin, 4E-BP2, and TRIM8 by using known interacting partner information, the affinity and off-targeting propensity of a partner protein-derived guide peptide are expected to vary based on the specific downselected peptide as well as properties of the partner protein, and future work to apply partner-derived guides to a greater number of targets will increase confidence in our approach.

Our work further demonstrates the application of pLMs to identify protein–protein binding interfaces, which while less effective on structured targets than current state-of-the-art approaches, are competitive with structural homology or feature-based methods. We envision that future models, which combine larger language models with evolutionary and structural information and directly consider both interacting proteins, may further improve performance both computationally and experimentally. In total, by integrating pLM-based binding predictions with uAbs for protein degradation, our work motivates the utilization of protein interactions to design programmable tools for broad-scale proteome editing applications.

## Methods

### Dataset generation

The PDB-derived dataset for this paper was generated by mining the RCSB PDB for verified, high-resolution PPI structures. Every interaction of every assembly of every co-crystal in the PDB was retrieved, and then the interactions were filtered for uniqueness (a unique interaction was one with a unique pair of partners, or with significantly different (>100 Å^2^) buried surface area for the same pair of partners). Filtration yielded 420,000 PPIs. Next, all interaction structures with amino acid sequence length greater than 50 and less than 1023 (for computational training speed) were processed with Rosetta PeptiDerive^13^, extracting a list of derived peptides and their associated Rosetta energy scores (REUs), with lower scores indicating higher predicted stability. After filtering entries with lower than -1000 REU, the REU scores for 10-mer peptides at each position were averaged to estimate a per amino acid position energy score. The per position energy score was averaged between matching derived protein sequences, so that the dataset does not include redundant entries. The energy score was thresholded at -1 and converted to a binary classification task with less than -1 energy being a protein binding amino acid and energy greater than -1 being a non-binding amino acid. Protein sequences were then clustered using MMseqs connected component clustering at 0.25 minimum sequence identity to group homologous proteins. Training, validation and testing sets were created with 26,423 train, 3487 validation, and 3817 test sequences, with no entries across different sets belonging to the same cluster. Thus, validation and test metrics do not reflect memorization of the properties of homologous protein sequences. Proteins which were clustered by MMseqs to partner proteins selected for in vitro testing were also moved to the test set. For benchmarking, the Dockground-based PPBS dataset used in ScanNet was utilized^12,28^. The dataset was processed into a sequence-to-binary target list format for the model.

### Interactome curation and calculations

The percentage of the human proteome with at least one binding partner was estimated by screening three databases: IMEx (https://www.imexconsortium.org/), BioGRID (https://thebiogrid.org/), or PROPER (https://genemo.ucsd.edu/proper/)^6–8^. Only databases that explicitly provide experimental evidence of physical binding were considered. This criterion excluded StringDB, which does not guarantee physical interaction. The gene symbols corresponding to each human protein were downloaded from UniProt (20601 total). For each database, pandas was used to scan for symbols and compile lists of proteins involved in at least one PPI. Screening was performed separately for heterogeneous interactions and homogeneous (self-binding) interactions. To account for varying curation standards, the entire process was repeated twice with different sets of filters. The most stringent or least inclusive filtering included PROPER entries with *p* < 0.01, all IMEx entries, and BioGRID entries justified by low throughput (LTP) physical evidence. The least stringent or most inclusive filtering included PROPER entries with *p* < 0.05, all IMEx entries, and BioGRID entries justified by either LTP or high throughput (HTP) physical evidence. Results are provided for both cases.

To quantify the availability of structural data on PPIs, the PDB was scanned for co-crystal complexes of two human proteins. Complexes were divided into two categories: heteromeric and homomeric. The PDB provides an Entry ID for each co-crystal and FASTA sequences for its two constituent proteins. Because species indications and constituent Entry IDs were not directly available, determining the co-crystal composition required a multistep process: (i) mapping co-crystal Entry IDs to organisms and filtering for human-human interactions only (reference: source.idx from the PDB archive, https://ftp.wwpdb.org/pub/pdb/derived_data/index/) (ii) mapping the constituent proteins in each co-crystal to Entry IDs based on their FASTA sequences (reference: pdb_seqres.txt from the PDB archive, https://ftp.wwpdb.org/pub/pdb/derived_data/) (iii) mapping Entry IDs to UniProt KB identifiers and UniProt gene symbols (references : SIFTS database pdb_chain_uniprot.csv, https://www.ebi.ac.uk/pdbe/docs/sifts/quick.html, UniProt Retrieve/ID Mapping tool) (iv) comparing the list of PDB-derived UniProt gene symbols to the full human genome. The final result represents the total number of human proteins involved in at least one co-crystal in PDB.

### Model training

The model is based upon Meta AI’s ESM-2 model (https://github.com/facebookresearch/esm) with a neural network head trained to classify the per amino acid interacting positions^9^. The final three layers of ESM-2 650 M were fine tuned together with a four layer fully connected neural network classification head which processes each position output of ESM-2 to predict a per position probability. Each protein is passed to the model with the per amino acid binary class as the target for cross entropy loss: −(*y*log(*p*) + (1 − *y*)log(1 − *p*)) where *y* is the binary class label (0 = nonbinding and 1 = binding) and *p* is the predicted probability of the amino acid belonging to a binding site. The model was implemented using PyTorch and trained until validation loss began to increase. When using the PPBS dataset, the weighting method used in Tubiana, et al., was adopted by multiplying the loss by the specified weight for for consistency with ScanNet^12^.

### Model evaluation

The model was evaluated and tested on held out binding partner proteins. Metrics are chosen to reflect the computational task (AUROC, Spearman correlation) and the downstream laboratory task (top *n* amino acid energy scores, % of top predicted amino acids meeting a minimum binding energy threshold).

### PPBS benchmarking

PPBS dataset, test splits, as well as model test scores were obtained directly from Tubiana, et al.^12^ The PPBS dataset splits represent: 70% (at least 70% sequence homology with one training example), homology (at most 70% homology with a train set example, although at least one train set belongs to the same protein superfamily), topology (at least one train set has a similar protein topology with none in the similar protein family) and None (none of the above groups). The structural homology baseline uses template protein chains with known binding sites, a local pairwise structural comparison, and an alignment weighting scheme. The handcrafted features baseline includes 58 features based on the structural, atomic, and sequence information, with an XGBoost algorithm. Note that MaSIF-site was not retrained for the per-residue task. Additional details and implementation of baseline models and test data splits can be found in Tubiana, et al.^12^ Comparisons between the predicted SaLT&PepPr scores and experimentally-annotated PPBS binding sites on different protein structures in the PPBS dataset were visualized in PyMol, with a red-to-blue color scheme. Red indicates high binding probability amino acids, with blue as low binding probability, normalized for each protein chain.

### Generation of plasmids

All uAb plasmids were generated from the standard pcDNA3 vector, harboring a cytomegalovirus (CMV) promoter and a C-terminal IRES-mCherry cassette. Target coding sequences (CDS) were synthesized as gBlocks from Integrated DNA Technologies (IDT). Sequences were amplified with overhangs for Gibson Assembly-mediated insertion into the pcDNA3-SARS-CoV-2-S-RBD-sfGFP backbone (Addgene #141184) linearized by digestion with NheI and BamHI. An Esp3I restriction site was introduced immediately upstream of the CHIPΔTPR CDS and flexible GSGSG linker via the KLD Enzyme Mix (NEB) following PCR amplification with mutagenic primers (Genewiz). For uAb assembly, oligos for candidate peptides were annealed and ligated via T4 DNA Ligase into the Esp3I-digested uAb backbone. Assembled constructs were transformed into 50 µL NEB Turbo Competent *Escherichia coli* cells, and plated onto LB agar supplemented with the appropriate antibiotic for subsequent sequence verification of colonies and plasmid purification (Genewiz). For protein purification, genes encoding each of the uAb constructs were PCR amplified from pcDNA3-based plasmids using primers that introduced HindIII and XhoI overhangs. The resulting PCR amplicons were ligated in an empty pET28a vector, which had been doubly digested with HindIII/XhoI. This process yielded plasmids which encoded each of the selected peptides followed by CHIPΔTPR, now bearing a 6xHis tag at its C-terminus. All plasmids were confirmed by DNA sequencing by Genewiz or at the Biotechnology Resource Center (BRC) Genomics Facility of the Cornell Institute of Biotechnology, and subjected to plasmid purification.

### Cell culture

The DLD1 cell line was a generous gift from Dr. Pengbo Zhou. DLD1 cells (ATCC CCL-221), HEK293T cells (ATCC CRL-3216), and A673 cells (ATCC CRL-1598) were cultured in DMEM supplemented with 100 units/mL penicillin, 100 mg/mL streptomycin, and 10% FBS. Unless otherwise noted, the day before the transfection, 0.3 × 10^6^ cells were seeded in each well of a 6-well plate. uAb-expressing plasmids were prepared using the PureYield miniprep kit to remove endotoxins. On the day of transfection, plasmids were transfected by Lipofectamine 3000. After 3 days of incubation post-transfection, cell lysates were collected for immunoblotting.

### Cell fractionation and immunoblotting

For probing β-catenin in Fig. 2, on the day of harvest, cells were detached by addition of 0.05% trypsin-EDTA and cell pellets were washed twice with ice-cold 1× PBS. Cells were then lysed and subcellular fractions were isolated from lysates using a Subcellular Protein Fractionation Kit (ThermoFisher) per the manufacturer’s instructions. Specifically, ice-cold cytosolic extraction buffer was added to the cell pellet, the mixture was placed at 4 °C for 10 min with gentle shaking followed by centrifugation at 500 × g for 10 min at 4 °C. The supernatant was collected immediately to a pre-chilled PCR tube and placed on ice followed by immunoblotting or stored at −20 °C for future usage. The pellet was then added with ice-cold membrane extraction buffer. The mixture was incubated at 4 °C for 10 min followed by centrifugation at 3000 × g for 5 min. The supernatant was immediately transferred to a pre-chilled tube. Protein concentration was quantified using the Pierce BCA Protein Assay Kit (ThermoFisher). An equivalent amount of total protein was loaded into Precise Tris-HEPES 4−20% sodium dodecyl sulfate (SDS)-polyacrylamide gels (ThermoFisher) and separated by electrophoresis. Immunoblotting was performed according to standard protocols. Briefly, proteins were transferred to poly(vinylidene fluoride) (PVDF) membranes (Millipore), blocked with 5% (w/v) nonfat dry milk (Carnation) in 1× tris-buffered saline (TBS) with 0.05% (v/v) Tween 20 (TBST) at room temperature for 1 h, washed three times with TBST for 10 min, and probed with rabbit anti-β-catenin antibody (Cell Signaling, Cat # 8480 S; diluted 1:1000) or rabbit anti-β-Tubulin (Cell Signaling Cat # 2146; diluted 1:1000). The blots were washed again three times with TBST for 5 min each and then probed with a secondary antibody, donkey anti-rabbit-horseradish peroxidase (HRP) (Abcam, Cat # 7083; diluted 1:2500), for 1 h at room temperature. Blots were detected by chemiluminescence using a ChemiDoc MP imager (Bio-Rad). Densitometry analysis of protein bands in immunoblots was performed using ImageJ software as described here: https://imagej.nih.gov/ij/docs/examples/dot-blot/. Briefly, bands in each lane were grouped as a row or a horizontal “lane” and quantified using ImageJ’s gel analysis function. Intensity data for the uAb bands was normalized to band intensity for empty plasmid control cases from six independent experiments.

For probing TRIM8 and 4E-BP2 in Fig. 3, on the day of harvest, cells were detached by addition of 0.05% trypsin-EDTA and cell pellets were washed twice with ice-cold 1× PBS. Cells were then lysed and subcellular fractions were isolated from lysates using a 1:100 dilution of protease inhibitor cocktail (Millipore Sigma) in Pierce RIPA buffer (ThermoFisher). Specifically, the protease inhibitor cocktail-RIPA buffer solution was added to the cell pellet, the mixture was placed at 4 °C for 30 min followed by centrifugation at 15,000 rpm for 10 min at 4 °C. The supernatant was collected immediately to a pre-chilled PCR tube, and after adding 4× Bolt™ LDS Sample Buffer (ThermoFisher) with 5% β-mercaptoethanol in a 3:1 ratio, the mixture was incubated at 95 °C for 10 min prior to immunoblotting. Immunoblotting was performed according to standard protocols. Briefly, samples were loaded at equal volumes into Bolt™ Bis-Tris Plus Mini Protein Gels (ThermoFisher) and separated by electrophoresis. iBlot™ 2 Transfer Stacks (Invitrogen) were used for membrane blot transfer, and following a 1 h room-temperature incubation in SuperBlock™ Blocking Buffer (ThermoFisher), proteins were probed with rabbit anti-TRIM8 antibody (Cell Signaling, Cat # 4936, diluted 1:500), rabbit anti-4E-BP2 antibody (Cell Signaling, Cat # 2845 T, diluted 1:500), rabbit anti-Vinculin antibody (ThermoFisher, Cat # 700062, diluted 1:500), or mouse anti-GAPDH (Santa Cruz Biotechnology, Cat # sc-47724; diluted 1:500) for overnight incubation at 4 °C. The blots were washed three times with 1× TBST for 5 min each and then probed with a secondary antibody, goat anti-rabbit IgG (H + L), horseradish peroxidase (HRP) (ThermoFisher, Cat # 31460, diluted 1:5000) or goat anti-mouse IgG (H + L) Poly-HRP (ThermoFisher, Cat # 32230, diluted 1:2000) for 1–2 h at room temperature. Following three washes with 1× TBST for 5 min each, blots were detected by chemiluminescence using an iBright 1500 Imaging System (ThermoFisher). Densitometry analysis of protein bands in immunoblots was performed using FIJI software as described here: https://imagej.nih.gov/ij/docs/examples/dot-blot/. Briefly, bands in each lane were grouped as a row or a horizontal “lane” and quantified using FIJI’s gel analysis function. Intensity data for the uAb bands was first normalized to band intensity of GAPDH (for TRIM8) or vinculin (for 4E-BP2) in each lane then to the average band intensity for empty uAb vector control cases across replicates.

### TOPFlash assay

A total of 1 × 10^4^ DLD1 cells were seeded on a white-bottom 96-well plate 20–24 h prior to transfection. On the day of transfection, each well received the following plasmids: M50 Super 8× TOPFlash plasmid (Addgene plasmid # 12456) or M51 Super 8× FOPFlash (TOPFlash mutant; Addgene plasmid # 12457), pCMV-Renilla^29^, and pcDNA3-SnP_7 or pcDNA3-SnP_8. A total of 100 ng of plasmid DNA in a ratio of TOPFlash/FOPFlash : Renilla : SnP_7/SnP_8 uAb = 1:0.1:3 was mixed with Lipofectamine 3000 reagent in serum free Opti-MEM medium and added dropwise to each well after incubation at room temperature for 15 min. After 48 h of incubation, cells were lysed and the firefly and Renilla luminescence signals were measured sequentially by the dual-luciferase reporter kit (Promega). Plates were read on a microplate reader (Tecan). The luciferase activities were measured and normalized against the control Renilla activities.

### Protein expression and purification

All purified uAb constructs, and unfused CHIPΔTPR were obtained from cultures of *E. coli* BL21(DE3) cells carrying pET28a-based plasmids encoding the SnP_7 the SnP_8 uAbs or CHIPΔTPR^3^. Cells were grown in Luria-Bertani (LB) medium according to protocols described previously^3^. Briefly, protein expression was induced with 1 M isopropyl β-D-1-thiogalactopyranoside (IPTG) when the culture density, determined by optical density at 600 nm (OD_600_), reached 0.5–0.7 and proceeded for 12–16 h at 37 °C. Following expression, cells were harvested by centrifugation at 10,000 × g for 10 min at 4 °C. The resulting pellets were resuspended in 10 mL of phosphate-buffered saline (PBS) and lysed using an EmulsiFlex-C5 high-pressure homogenizer (Avestin). Lysates were cleared of insoluble material by centrifugation at 10,000 × g for 10 min at 4 °C. Clarified lysates containing 6xHis-tagged proteins were subjected to gravity-flow Ni^2+^-affinity purification using HisPur Ni-NTA resin (ThermoFisher) following the manufacturer’s protocols. Purified proteins were stored at 4 °C for up to 2 weeks. The final purity of all proteins was confirmed by Coomassie-blue staining of SDS-PAGE gels.

### ELISA

ELISA was performed according to previously published protocols^3^. Briefly, 96-well plates (MaxiSorp; Nunc Nalgene) were incubated with 1 μg/mL of β-catenin (Biomatik, Cat # RPU40704) diluted in PBS, pH 7.4, 50 µL/well, at 4 °C overnight. Plates were incubated with 200 µL blocking buffer (5% (w/v) nonfat dry milk (Carnation) in PBS) overnight at 4 °C, then washed three times with 200 µL PBS-T (PBS, 0.1% (v/v) Tween 20) per well. Purified uAb constructs were biotinylated with EZ-Link™ NHS-Biotin (ThermoFisher, Cat # 20217) following the manufacturer’s instructions. The biotinylated uAb constructs were appropriately serially diluted in triplicate in PBS and added to the ELISA plates for 1 h at 37 °C. Plates were washed three times with PBS-T, then incubated for 1 h at room temperature in the presence of HRP-conjugated streptavidin (ThermoFisher, Cat # N100; diluted 1:20,000), with shaking at 450 rpm. After another three PBS-T washes, 100 µL of 3,3’-5,5’-tetramethylbenzidine substrate (1-Step Ultra TMB-ELISA; ThermoFisher) was added to each well, and the plates were incubated at room temperature in the dark. The reaction was stopped by adding 100 µL of 2 M H2SO4, and absorbance was measured at a wavelength of 450 nm using a FilterMax F5 microplate spectrophotometer (Agilent).

### Proteomics

HEK293T cells were maintained in DMEM supplemented with 100 units/mL penicillin, 100 mg/mL streptomycin, and 10% FBS. Target-sfGFP (1 µg) and Target-sfGFP (1 µg) + pcDNA-uAb (1 µg) plasmids were transfected into cells as triplicates (8 × 10^4^/well in a 6-well plate) with Lipofectamine 3000 (Invitrogen) in Opti-MEM (Gibco). Three days post transfection, cells were harvested and washed four times with 500 µL 1X cold PBS. The cell pellets were resuspended in 200 µL Pierce RIPA buffer (VWR) and incubated on ice for 15 min. The homogenates were treated with 20% (w/v) SDS in triethylammonium bicarbonate buffer, pH 8.5, followed by probe sonication and heating at 80 °C for 5 min. The supernatants were collected after centrifugation and the concentrations were determined using detergent-compatible Bradford assay. From each sample, 20 µg was reduced and alkylated, and digested with trypsin using an S-trap micro device. Peptide eluents were lyophilized, and after reconstitution, equal volumes of each sample were mixed to make an SPQC pool. Approximately 1 µg of each sample, and three replicates of the SPQC pool were analyzed by 1D-LCMS/MS. Samples were analyzed using a M-Class UPLC system (Waters) coupled to an Exploris 480 high resolution accurate tandem mass spectrometer (ThermoFisher) via a Nanospray Flex Ion source and processed using Spectronaut 16. The *p* values were calculated by performing a Student’s *t*-test on log_2_fc values. The log_2_fc values were calculated by the difference of average abundances of the proteins in the presence and absence of uAb.

### Functional assays

For the apoptosis assay, 3 × 10^5^ A673 cells/well were seeded on a 24-well plate 20–24 h prior to transfection. On the day of transfection, each well received the following plasmids: ZipGFP-Casp3 plasmid (Addgene plasmid #81241) and pcDNA3-SnP_TRIM8_#. A total of 500 ng of plasmid DNA in a ratio of ZipGFP-Casp3:pcDNA3-SnP_TRIM8_# = 1:1 was mixed with Lipofectamine 2000 reagent in serum-free Opti-MEM medium and added dropwise to each well after incubation at room temperature for 20 min. After 60 h of incubation, cells were harvested and analyzed similarly as mentioned for uAb screening. Cells expressing mCherry were gated, and normalized EGFP cell fluorescence was calculated as compared to a sample transfected with a non-targeting uAb, using the FlowJo software (https://flowjo.com/). An example gating strategy is found in Supplementary Fig. 4.

### Statistics and reproducibility

To ensure robust reproducibility of all results, experiments were performed with at least three biological replicates and at least three technical measurements. Sample sizes were not predetermined based on statistical methods but were chosen according to the standards of the field (at least three independent biological replicates for each condition), which gave sufficient statistics for the effect sizes of interest. All data were reported as average values with error bars representing standard deviation (SD). For individual samples, unless described otherwise, statistical significance was determined by paired Student’s *t* tests (*p < 0.05, **p < 0.01; ***p < 0.001; ****p < 0.0001). All graphs were generated using Prism 9 for MacOS version 9.2.0. No data were excluded from the analyses. The experiments were not randomized. The investigators were not blinded to allocation during experiments and outcome assessment.

### Reporting summary

Further information on research design is available in the Nature Portfolio Reporting Summary linked to this article.

### Supplementary information

Supplemental Information

Reporting Summary

## Data Availability

All data needed to evaluate the conclusions in the paper are present in the paper and supplementary tables and figures. All raw and processed data, including peptide sequences, original immunoblots, and numerical data underlying figures, have been deposited to the Zenodo repository: 10.5281/zenodo.10008581^30^. Additional raw and processed proteomics data, and spectral library files, are available at the ProteomeXchange Consortium (PXD046088) via the MassIVE repository (ftp://MSV000093088@massive.ucsd.edu). Uncropped and unedited blot/gel images are included in Supplementary Figure 5. Example uAb expression plasmids can be found on Addgene (#101800 and #101801).

## Code availability

SaLT&PepPr training data and SaLT&PepPr code can be found at: https://github.com/programmablebio/saltnpeppr, which includes an easy-to-use Colab notebook for peptide generation.
