# Supplementary material for: SaLT&PepPr is an interface-predicting language model for designing peptide-guided protein degraders
Source: Commun Biol. 2023 Oct 24;6:1081. doi: 10.1038/s42003-023-05464-z (PMC10598214; doi:10.1038/s42003-023-05464-z)
Supplement: Supplementary file 1 — Supplemental Information [file 42003_2023_5464_MOESM1_ESM.pdf]

Supplementary Information

SaLT&PepPr is an Interface-Predicting Language Model for Designing Peptide-Guided Protein Degraders

Garyk Brixi,[1,*] Tianzheng Ye,[2,*] Lauren Hong,[1,*] Tian Wang,[1] Connor Monticello,[3] Natalia Lopez-Barbosa,[2] Sophia Vincoff,[1] Vivian Yudistyra,[1] Lin Zhao,[1] Elena Haarer,[1] Tianlai Chen,[1] Sarah Pertsemlidis,[1] Kalyan Palepu,[1] Suhaas Bhat,[1] Jayani Christopher,[1] Xinning Li,[1] Tong Liu,[1] Sue Zhang,[1] Lillian Petersen,[1] Matthew P. DeLisa[2,3,4] and Pranam Chatterjee[1,5,6,†]

1. Department of Biomedical Engineering, Duke University
2. Robert F. Smith School of Chemical and Biomolecular Engineering, Cornell University
3. Meinig School of Biomedical Engineering, Cornell University
4. Cornell Institute of Biotechnology, Cornell University
5. Department of Computer Science, Duke University
6. Department of Biostatistics and Bioinformatics, Duke University

*These authors contributed equally
†Corresponding author: pranam.chatterjee@duke.edu

Supplementary Figures

1. PDB-Derived dataset generation for model training.
2. Inference times across interface-aware peptide derivation methods.
3. Validation of β-catenin degradation via mass spectrometry.
4. Example gating strategy for flow cytometry analysis.
5. Unedited/uncropped immunoblots.

Supplementary Tables

1. SaLT&PepPr-derived peptide sequences and scores.

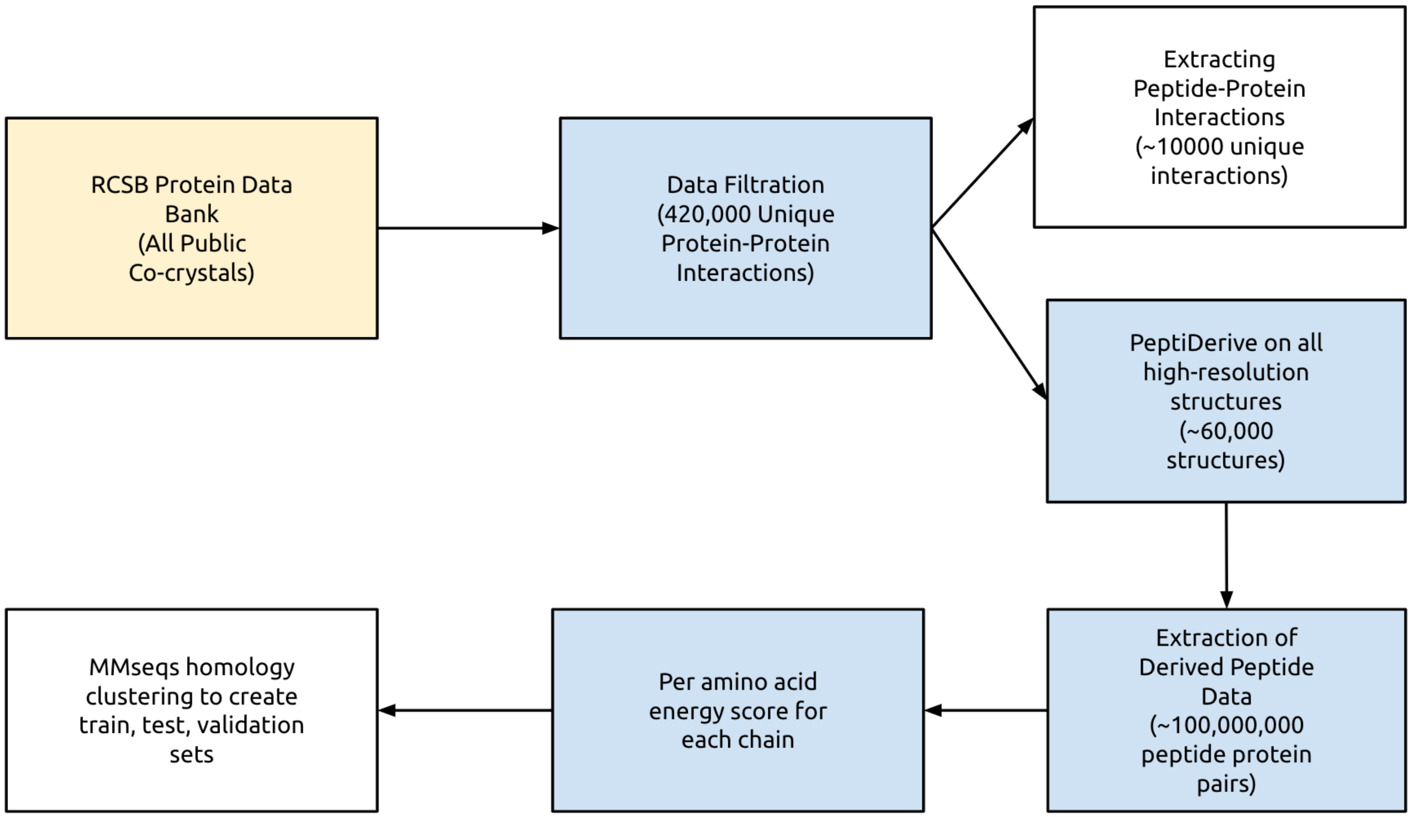

Supplementary Figure 1. PDB-derived dataset generation for model training. The RCSB Protein Data Bank was mined for verified, high-resolution PPI structures. Every interaction of every assembly of every co-crystal in the PDB was filtered for uniqueness (a unique pair of partners or >100 Å^2 buried surface area for the same pair of partners), yielding 420,000 PPIs. Next, interaction structures were processed with PeptiDerive, extracting a list of derived peptide "hot sequences", and their associated Rosetta energy unit (REU) scores on a per amino acid basis. Homology clustering was conducted for training and validation set derivation.

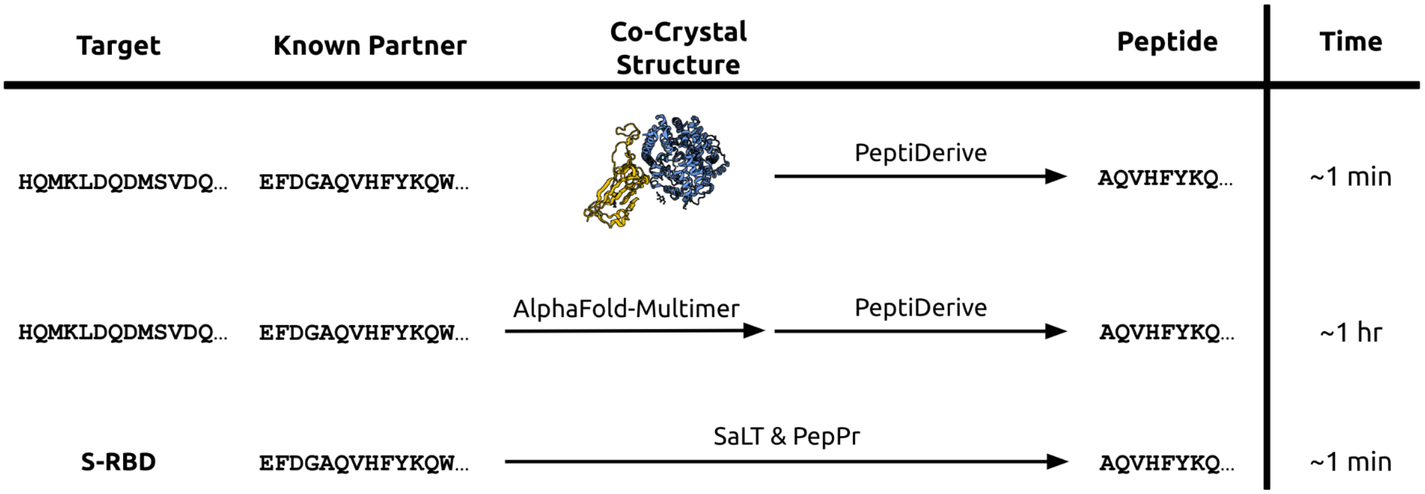

Supplementary Figure 2. Inference times across interface-aware peptide derivation methods. Observed average times for inference using indicated methods are shown under “Time”. A standard machine with 2 CPU cores, 8 GB of RAM, and no GPU was used for inference.

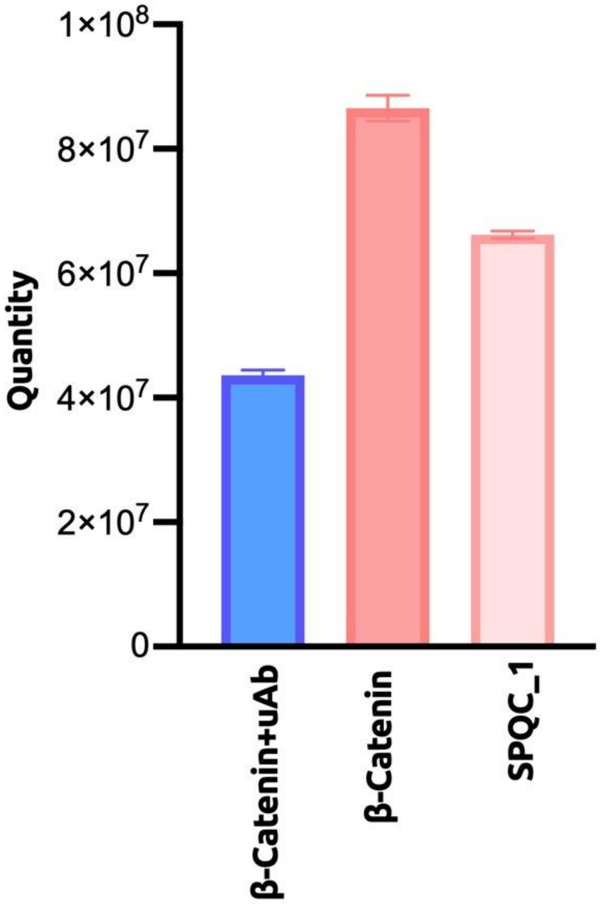

Supplementary Figure 3. Validation of β-catenin degradation via mass spectrometry. The abundances of β-Catenin (CTNNB1) were analyzed in triplicates with 1D-LCMS/MS in the presence and absence of the uAb and the SPQC pool. The average abundance of CTNNB1 was calculated for the triplicates and plotted using GraphPad Prism.

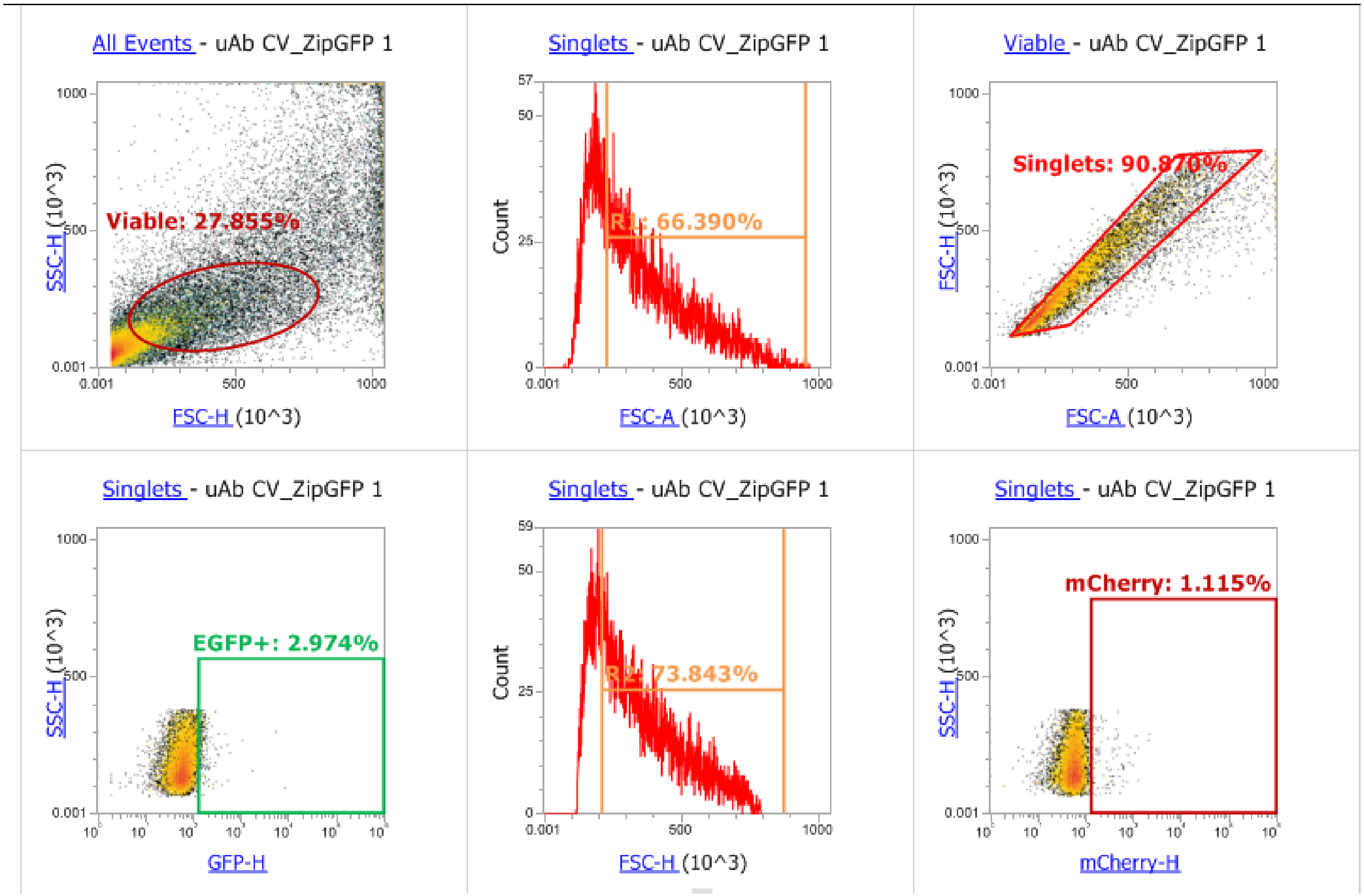

Supplementary Figure 4. Example gating strategy for flow cytometry. ~10,000 gated events for data analysis based on the default FSC/SSC parameters for A673 cells. The GFP+ and mCherry+ gates were established both by a GFP- negative control and an mCherry- control. All analysis was conducted in FlowJo.

Supplementary Figure 5. Unedited/uncropped immunoblots.

	Anti-4E-BP2	Anti-Vinculin
Replicate 1 (left) + Replicate 2 (right) (Vinculin top, 4E-BP2 bottom)	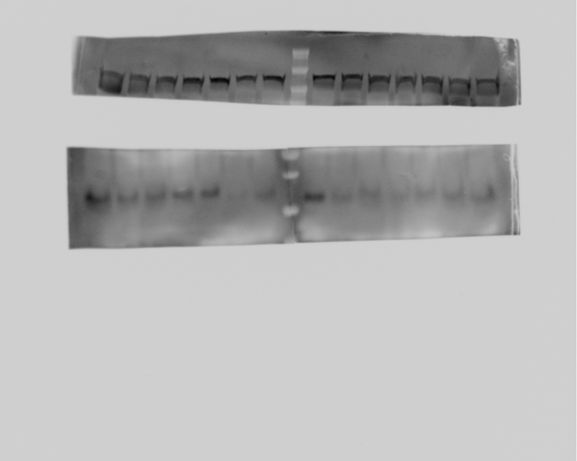	
Replicate 3	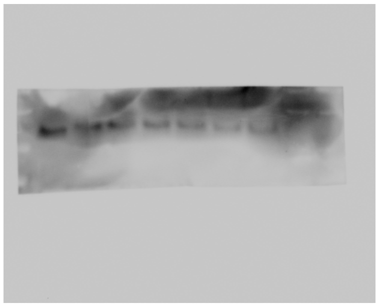	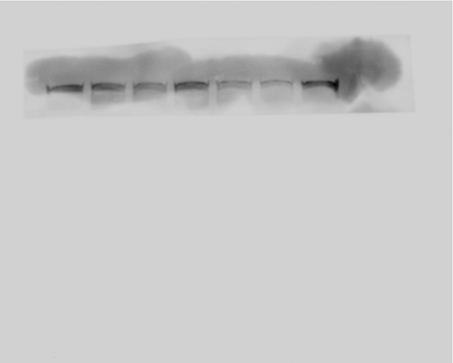

	Anti-TRIM8	Anti-GAPDH
Replicate 1 (TRIM8 top, GAPDH bottom)	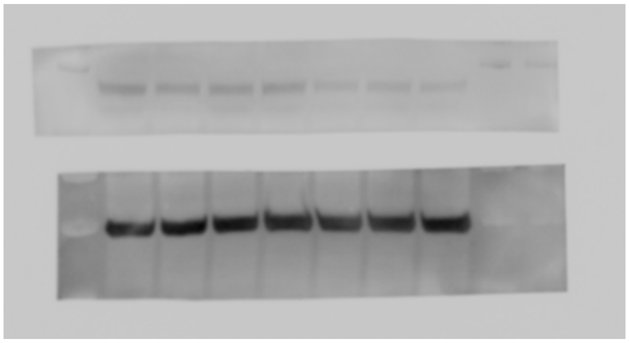	

Replicate 2 (TRIM8 top, GAPDH bottom)	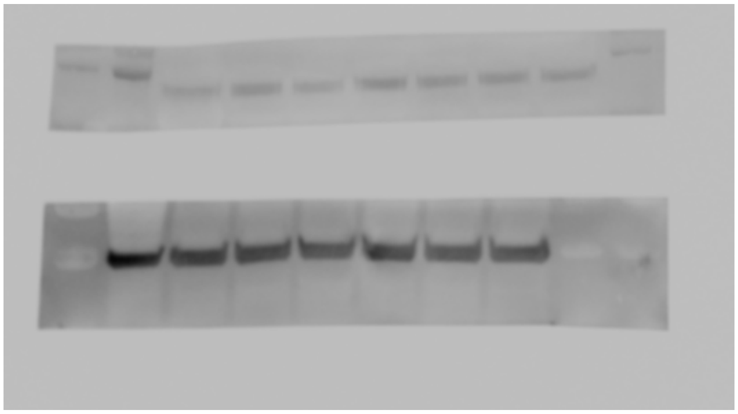	
Replicate 3	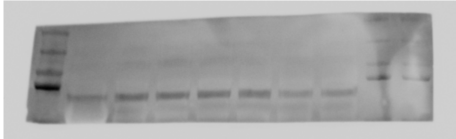	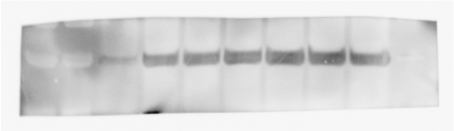

	Anti-β-catenin	Anti-β-Tubulin
	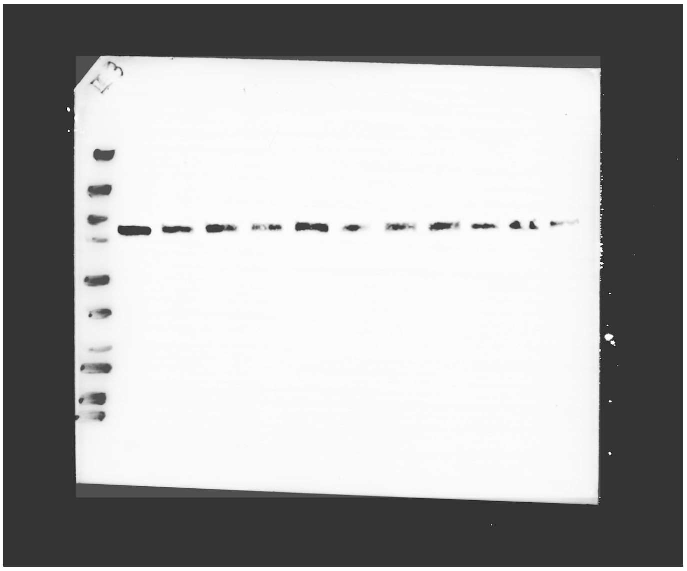	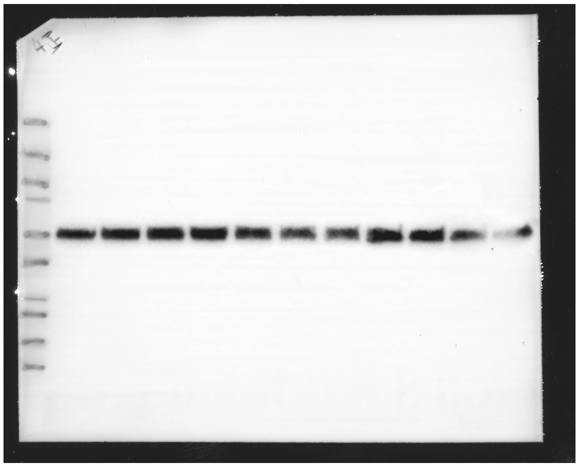

Supplementary Table 1. SaLT&PepPr-derived peptide sequences and scores. Peptides were built per target protein based on specific binders using SaLT&PepPr (SnP) with provided scores indicating the cumulative binding site probability of the peptide. These were cloned into uAb constructs for subsequent analysis via flow cytometry.

Target	Binder Name or UniProt ID	Peptide Name	Sequence	SnP Score
β-catenin	CADH1	β-cat_SnP_1	DYEGSGSEAASLSSLNSSESDKDQ	0.509226
β-catenin	CADH1	β-cat_SnP_2	YDSLLVFDYE	0.39451852
β-catenin	CADH1	β-cat_SnP_3	AADTDPTAPPYDSLLVFDYE	0.36254913
β-catenin	CADH1	β-cat_SnP_4	PTAPPYDSLLVFDYE	0.38101247
β-catenin	CADH1	β-cat_SnP_5	YDSLLVFDYEG	0.40408888
β-catenin	CADH1	β-cat_SnP_6	TAPPYDSLLVFDYE	0.38535473
β-catenin	CADH1	β-cat_SnP_7	DPTAPPYDSLLVFDYEGS	0.39557245
β-catenin	CADH1	β-cat_SnP_8	PTAPPYDSLLVFDYEG	0.38843626
4E-BP2	eIF4E	4E-BP2_SnP_1	LETLLCLIGESF	0.36761266
4E-BP2	eIF4E	4E-BP2_SnP_2	LSSNLMPGCDYS	0.52337885
4E-BP2	eIF4E	4E-BP2_SnP_3	KNDKSKTWQANL	0.5253146
4E-BP2	eIF4E	4E-BP2_SnP_4	ALWFFKNDKSKT	0.5697511
4E-BP2	eIF4E	4E-BP2_SnP_5	DFWALYNHIQLS	0.6841912
4E-BP2	eIF4E	4E-BP2_SnP_6	KFDTVEDFWALY	0.68449545
TRIM8	P03372	TRIM8_SnP_1	LDKITDTLIHLM	0.9952535
TRIM8	O00463	TRIM8_SnP_2	MRLVLEKNVQLE	0.9897295
TRIM8	P03372	TRIM8_SnP_3	LLLILSHIRHMS	0.98955286
TRIM8	G4XH65	TRIM8_SnP_4	LAQLLLILSHIR	0.9862616
TRIM8	D6R9G5	TRIM8_SnP_5	SPYFHAMFTGEM	0.984835
TRIM8	Q9Y5K6	TRIM8_SnP_6	IVEALKKDHGKE	0.97291374
