# Supplementary material for: SaLT&PepPr is an interface-predicting language model for designing peptide-guided protein degraders
Source: Commun Biol. 2023 Oct 24;6:1081. doi: 10.1038/s42003-023-05464-z (PMC10598214; doi:10.1038/s42003-023-05464-z)
Supplement: Supplementary file 2 — Reporting Summary [file 42003_2023_5464_MOESM2_ESM.pdf]

nature research

Corresponding author(s): Pranam Chatterjee

Last updated by author(s): Oct 11, 2023

Reporting Summary

Nature Research wishes to improve the reproducibility of the work that we publish. This form provides structure for consistency and transparency in reporting. For further information on Nature Research policies, see our Editorial Policies and the Editorial Policy Checklist.

Statistics

For all statistical analyses, confirm that the following items are present in the figure legend, table legend, main text, or Methods section.

n/a	Confirmed	
☐	☒	The exact sample size (*n*) for each experimental group/condition, given as a discrete number and unit of measurement
☐	☒	A statement on whether measurements were taken from distinct samples or whether the same sample was measured repeatedly
☐	☒	The statistical test(s) used AND whether they are one- or two-sided<br>*Only common tests should be described solely by name; describe more complex techniques in the Methods section.*
☒	☐	A description of all covariates tested
☒	☐	A description of any assumptions or corrections, such as tests of normality and adjustment for multiple comparisons
☐	☒	A full description of the statistical parameters including central tendency (e.g. means) or other basic estimates (e.g. regression coefficient) AND variation (e.g. standard deviation) or associated estimates of uncertainty (e.g. confidence intervals)
☒	☐	For null hypothesis testing, the test statistic (e.g. *F*, *t*, *r*) with confidence intervals, effect sizes, degrees of freedom and *P* value noted<br>*Give P values as exact values whenever suitable.*
☒	☐	For Bayesian analysis, information on the choice of priors and Markov chain Monte Carlo settings
☒	☐	For hierarchical and complex designs, identification of the appropriate level for tests and full reporting of outcomes
☒	☐	Estimates of effect sizes (e.g. Cohen's *d*, Pearson's *r*), indicating how they were calculated

Our web collection on statistics for biologists contains articles on many of the points above.

Software and code

Policy information about availability of computer code

Data collection	The dataset for this paper was generated by mining the RCSB Protein Data Bank (PDB) for verified, high-resolution protein-protein interaction structures. Every interaction of every assembly of every co-crystal in the PDB was retrieved, and then the interactions were filtered for uniqueness (a unique interaction was one with a unique pair of partners, or with significantly different (>100 Å2) buried surface area for the same pair of partners). Filtration yielded 420,000 protein-protein interactions (PPIs). Next, all interaction structures with amino acid sequence length greater than 50 and less than 1023 (the maximum input size of ESM) were processed with Rosetta Peptiderive, extracting a list of derived peptide “hot-sequences”, and their associated Rosetta energy scores (REUs), with lower scores indicating higher predicted stability. Finally, for each target protein, its derived peptides and their scores constituted a set of data points for the regression model, to enable the model to distinguish degraders from non-degraders for any given target.
Data analysis	Briefly, bands in each lane were grouped as a row or a horizontal “lane” and quantified using ImageJ’s gel analysis function. Intensity data for the uAb bands was normalized to band intensity for empty plasmid control cases from six independent experiments.<br>For mass spectrometry, The p values were calculated by performing a Student’s t-test on log2fc values. The log2fc values were calculated by the difference of average abundances of the proteins in the presence and absence of uAb.

For manuscripts utilizing custom algorithms or software that are central to the research but not yet described in published literature, software must be made available to editors and reviewers. We strongly encourage code deposition in a community repository (e.g. GitHub). See the Nature Research guidelines for submitting code & software for further information.

Data

Policy information about availability of data

All manuscripts must include a data availability statement. This statement should provide the following information, where applicable:

- Accession codes, unique identifiers, or web links for publicly available datasets
- A list of figures that have associated raw data
- A description of any restrictions on data availability

All data needed to evaluate the conclusions in the paper are present in the paper and supplementary tables and figures. All raw and processed data, including peptide sequences, original immunoblots, and numerical data underlying figures, have been deposited to the Zenodo repository: https://doi.org/10.5281/zenodo.8355610. Uncropped and unedited blot/gel images are included in Supplementary Figure 5. Example uAb expression plasmids can be found on Addgene (#101800 and #101801).

Field-specific reporting

Please select the one below that is the best fit for your research. If you are not sure, read the appropriate sections before making your selection.

☒ Life sciences ☐ Behavioural & social sciences ☐ Ecological, evolutionary & environmental sciences

For a reference copy of the document with all sections, see nature.com/documents/nr-reporting-summary-flat.pdf

Life sciences study design

All studies must disclose on these points even when the disclosure is negative.

Sample size	No sample size calculation was performed.
Data exclusions	Original target-sfGFP degradation data was excluded as pre-treatment sfGFP levels were non-reproducible between independent replication efforts.
Replication	All samples were performed in independent biological duplicates.
Randomization	No randomization is required for this study.
Blinding	Blinding is not required for this study.

Reporting for specific materials, systems and methods

We require information from authors about some types of materials, experimental systems and methods used in many studies. Here, indicate whether each material, system or method listed is relevant to your study. If you are not sure if a list item applies to your research, read the appropriate section before selecting a response.

Materials & experimental systems

n/a	Involved in the study
☐	☒ Antibodies
☐	☒ Eukaryotic cell lines
☒	☐ Palaeontology and archaeology
☒	☐ Animals and other organisms
☒	☐ Human research participants
☒	☐ Clinical data
☒	☐ Dual use research of concern

Methods

n/a	Involved in the study
☒	☐ ChIP-seq
☒	☐ Flow cytometry
☒	☐ MRI-based neuroimaging

Antibodies

Antibodies used	Anti-β-catenin antibody (Cell Signaling, Cat # 8480S; diluted 1:1,000) , rabbit anti-β-Tubulin (Cell Signaling Cat # 2146; diluted 1:1,000), anti-TRIM8 antibody (Cell Signaling, Cat # 4936, diluted 1:500), rabbit anti-4E-BP2 antibody (Cell Signaling, Cat # 2845T, diluted 1:500), rabbit anti-Vinculin antibody (ThermoFisher, Cat # 700062, diluted 1:500), mouse anti-GAPDH (Santa Cruz Biotechnology, Cat # sc-47724; diluted 1:500)
Validation	Antibodies were validated in the cell lines that they were originally validated in by the manufacturer, as well as in the cell lines used in this study prior to experimental testing.

Eukaryotic cell lines

Policy information about cell lines

Cell line source(s)	293T ATCC CRL-3216, A-673 ATCC CRL-1598, DLD1 ATCC CCL-221
Authentication	Key genomic sequences were PCR amplified and verified before conducting experiments.
Mycoplasma contamination	Cell lines were tested negative for mycoplasma contamination.
Commonly misidentified lines (See ICLAC register)	None used in this study.
